# Argonaute1‐Dependent LtmilR2 Negatively Regulated Infection of *Lasiodiplodia theobromae* by Targeting a Guanine Nucleotide Exchange Factor in RAS Signalling

**DOI:** 10.1111/pce.70058

**Published:** 2025-07-13

**Authors:** Caiping Huang, Yuanyuan He, Wei Zhang, Jingpeng Wang, Mei Liu, Borala Liyanage D. Deepali, Jiye Yan

**Affiliations:** ^1^ Beijing Key Laboratory of Environment Friendly Management on Fruit Diseases and Pests in North China, Institute of Plant Protection, Beijing Academy of Agriculture and Forestry Sciences Beijing China

**Keywords:** grapevine canker disease, *Lasiodiplodia theobromae*, milRNA, pathogenicity, RNA fungicide

## Abstract

*Lasiodiplodia theobromae* (*L. theobromae*) is the causative agent of grapevine canker disease, which is a serious threat to global grape production. Currently, no effective fungicides are available to manage this disease. In this study, LtmilR2 was identified as an Argonaute1 (AGO1)‐dependent small RNA produced by *L. theobromae*. Functional analysis revealed that both LtAGO1 and LtmilR2 negatively regulated the pathogenicity of *L. theobromae*. Through degradome sequencing, we identified *LtRASGEF*—a guanine nucleotide exchange factor involved in RAS signalling—as the target gene of LtmilR2. LtmilR2 suppressed the expression of *LtRASGEF*. Pathogenicity assays demonstrated that LtRASGEF was essential for fungal virulence. Furthermore, following infection, the expression of LtmilR2 rapidly decreased, while *LtRASGEF* expression quickly increased and positively regulated infection. Finally, we observed that the growth of *L. theobromae* was inhibited when an RNA duplex targeting LtmilR2 was delivered via the star polycation (SPc) nanocarrier, suggesting that LtmilR2 may serve as an RNA‐based fungicide target for grapevine canker disease management.

## Summary statement

- A potential RNA fungicide to treat grapevine canker diseases by targeting genes in *Lasiodiplodia theobromae*.

## 1 Introduction

Grapevine canker disease, caused by *Lasiodiplodia theobromae* (*L. theobromae*), has led to significant yield losses in the grape industry. The whole genome of *L. theobromae* was sequenced by Yan et al. (2013), and several effectors that influence the pathogen's virulence have since been identified (Huang et al. 2023; Thilini Chethana et al. 2020; Xing et al. 2023). Additionally, microbiome research has contributed to the control of grapevine stem diseases (Li et al. 2023). However, as an endophyte, the mechanisms by which *L. theobromae* establishes latent infections remain unknown. Currently, there are no highly resistant grapevine varieties or effective fungicides available to control grapevine canker disease. Understanding the pathogenesis of *L. theobromae* is therefore essential for developing effective preventive and control products. Given that *L. theobromae* pathogenicity is highly influenced by environmental factors, research has shown that fungal gene expression may be regulated at the post‐transcriptional level to adapt to changing conditions (Song et al. 2019).

MicroRNAs (miRNAs) are key regulators of post‐transcriptional gene expression in plants and animals, representing a class of endogenous small RNAs (sRNAs) of 20–24 base pairs (bp). miRNAs regulate target mRNA expression by inhibiting translation or translation inhibition and transcriptional silencing (Ghildiyal and Zamore 2009). In fungi, sRNAs similar to plant and animal miRNAs, termed microRNA‐like RNAs (milRNAs), have been identified and may play a role in regulating latent pathogenicity in *L. theobromae*, underscoring their potential as targets for innovative control strategies. While sRNAs are generated in plants and animals through the RNA interference (RNAi) pathway, involving proteins such as Dicer or Dicer‐like (DCL) proteins and RNA‐dependent RNA polymerase (RDR) (Fagard et al. 2000; Mallory and Vaucheret 2006), the sRNA biogenesis mechanism in fungi is notably more complex. For instance, fungal small interfering RNAs (siRNAs) can be produced via either DCL‐dependent or DCL‐independent pathways (Lee et al. 2010). In *Neurospora crassa* (*N. crassa*), AGO proteins function as slicers and are crucial for the generation of sRNAs, including milRNAs (Dang et al. 2014). However, limited research is available on sRNA generation, as well as the functions of sRNAs in fungal pathogenicity, highlighting a need for further investigation in this area.

Recent studies have revealed that certain milRNAs are instrumental in regulating fungal growth and development to facilitate pathogenicity. For instance, in *Sclerotinia sclerotiorum* (*S. sclerotiorum*), 44 milRNAs were found to be potentially associated with sclerotial development, with Ssc‐milR‐240 specifically linked to this process by epigenetically regulating its target, a histone acetyltransferase (Xia et al. 2020; Zhou et al. 2012). In *Puccinia striiformis* (the wheat stripe rust pathogen), a single milRNA with 85 predicted target genes has been identified. These targets are involved in metabolism, cell structure, transcriptional regulation and transport pathways (Feng et al. 2016). Additionally, the FoQDE2‐dependent milR‐87 in *Fusarium oxysporum* f. sp. *cubense* (Foc) targets a glycosyl hydrolase‐coding gene, significantly repressing its expression early in infection; knockdown of this target gene resulted in enhanced sporulation and pathogenicity of Foc (Li et al. 2022). Other milRNAs in fungal pathogens play similar roles in modulating virulence. For example, Vm‐milR1 in *Valsa mali* (*V. mali*) epigenetically suppresses its target gene to reduce virulence (Jin et al. 2019), while Vm‐milR16 specifically represses genes involved in pathogenicity, facilitating infestation (M. Xu et al. 2020).

In cross‐kingdom interactions, fungal milRNAs have been shown to enhance virulence by targeting host genes. For instance, Pst‐milR1 in wheat stripe rust suppresses the wheat *PR2* gene to increase pathogen virulence (B. Wang et al. 2017), and Vm‐milR1 in *V. mali* acts as an effector to silence a receptor kinase gene in apple, weakening the host immune response and promoting infection (M. Xu et al. 2022). Despite these advances, research on fungal milRNAs remains limited compared to miRNA studies in plants and animals. Specifically, no studies have yet explored milRNAs in *L. theobromae*, the causative agent of grapevine canker disease. There is no available biofungicide for control this disease, which is highlighting an urgent need for further research in this area.

In this study, we investigated the role of AGO1‐dependent milRNAs in the pathogenicity of *L. theobromae*, focusing specifically on LtmilR2. Expression of LtmilR2 was significantly reduced in the *LtAGO1* mutant, and functional analysis indicated that LtmilR2 negatively regulates the pathogenicity of *L. theobromae*. Through degradome sequencing, a guanine nucleotide exchange factor (GEF) involved in RAS signalling, LtRASGEF, was predicted as a target gene of LtmilR2. To confirm this, LtRASGEF‐GFP was co‐expressed in *Nicotiana benthamiana* (*N. benthamiana*) leaves with or without LtmilR2. The results showed that leaves expressing only LtRASGEF‐GFP exhibited much stronger fluorescence than those co‐expressing LtRASGEF‐GFP with LtmilR2, indicating that LtmilR2 suppresses *LtRASGEF* expression. Additionally, knockout of the *LtRASGEF* gene in *L. theobromae* resulted in reduced pathogenicity. These findings suggest that LtmilR2 targets the LtRASGEF gene to enhance *L. theobromae* pathogenicity.

## 2 Results

### 2.1 *LtAGO1* Is Involved in the Mycelial Growth and Virulence of *L. theobromae*

**Figure 1:**
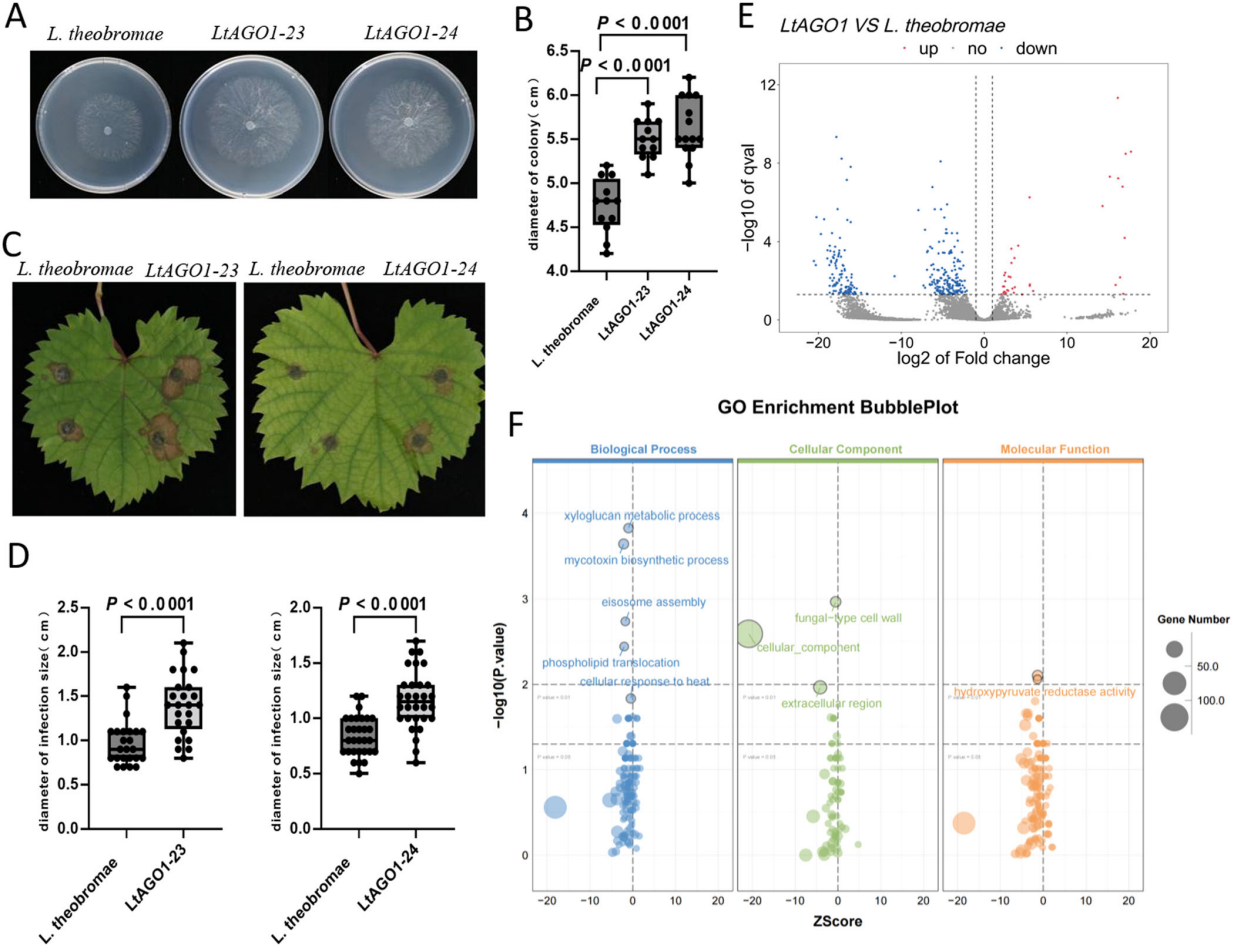
Pathogenicity analysis and transcriptome analysis of *LtAGO1* mutants. (A) Colony morphology of wild‐type *L. theobromae* and *LtAGO1* mutant strains (#23 and #24) after 24 h of growth on PDA medium. (B) Colony diameter was measured (*n* > 8), and statistical analysis was conducted using *t*‐test (*p* < 0.0001). (C) Pathogenicity assay of *LtAGO1* mutants on detached leaves of *Vitis vinifera* cv. Summer Black. (D) Lesion size was measured 3 days post‐inoculation (*n* > 20) (*t*‐test, *p* < 0.0001). (E) Analysis of transcriptome expression difference between *LtAGO1* mutant (#23) and wild type after 48 h of growth on PD medium with grape bark powder (Fold change (FC) ≥ 2 or ≤ 0.5, *p* < 0.05). (F) GO enrichment analysis of genes. Data are means ± SD for three replicates.

To elucidate the role of AGO1‐dependent milRNAs in the pathogenicity of *L. theobromae*, we conducted an orthologous protein BLAST and phylogenetic analysis. Four AGO proteins were identified in *L. theobromae* and they clustered with AGO proteins from other fungi, such as *N. crassa* and *V. mali*, as anticipated (Supporting Information S1: Figure 1A). To ascertain which AGO protein is involved in the biosynthesis of sRNAs in *L. theobromae*, we knocked out *AGO1* genes. The *LtAGO1* mutants were confirmed using four pairs of primers through PCR (Supporting Information S1: Figure 1B).

**Figure 2:**
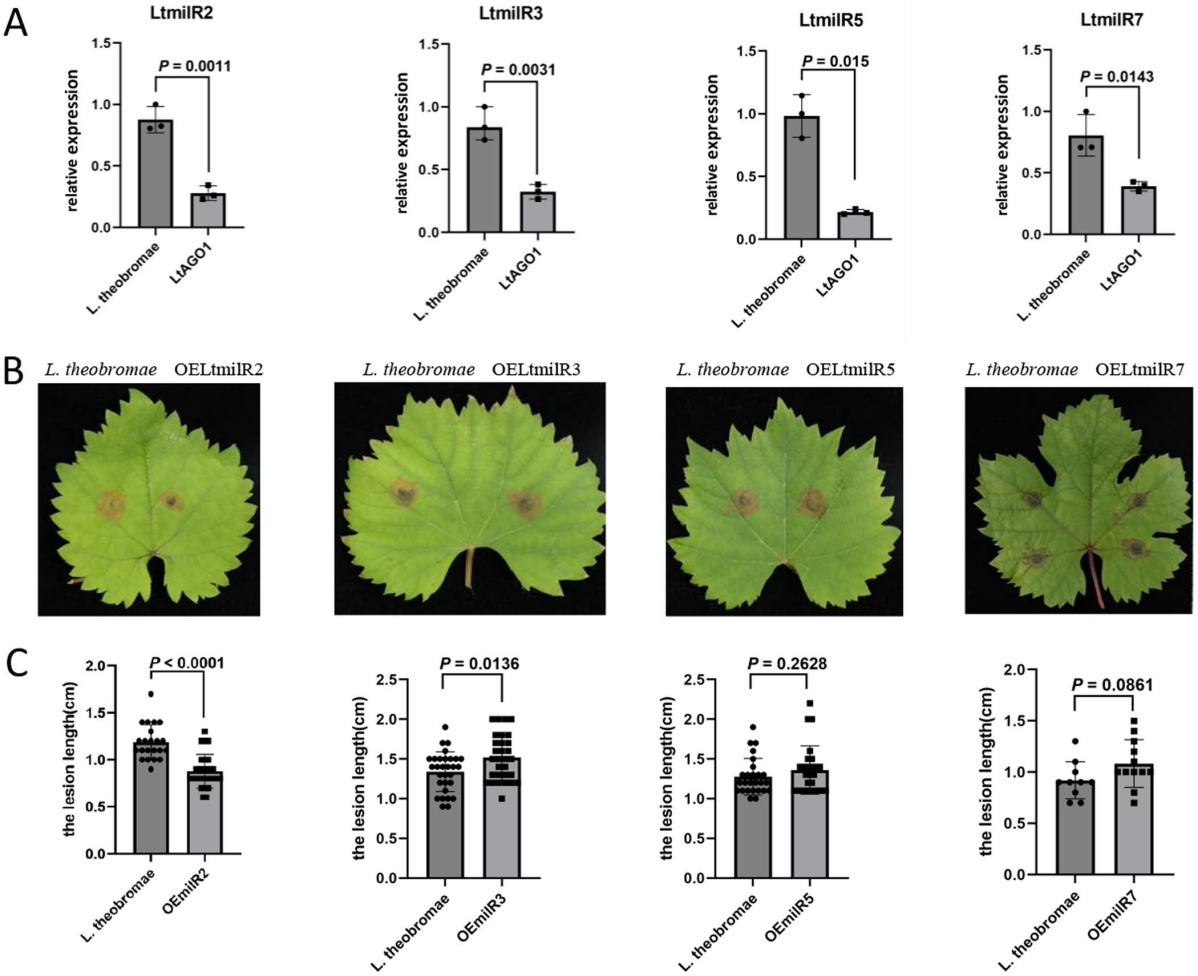
LtmilR2 was identified as an AGO1‐dependent milRNA. (A) Expression analysis of candidate *LtAGO1*‐dependent milRNAs by stem loop RT‐PCR and statistical analysis was conducted using two‐sided *t*‐test. (B) Pathogenicity assay of OEmilR2, OEmilR3, OEmilR5, OEmilR7 on detached leaves of *Vitis vinifera* cv. Summer Black. (C) Lesion size was measured 3 days post‐inoculation (*n* > 20), with significance assessed by two‐sided *t*‐test. [Color figure can be viewed at wileyonlinelibrary.com]

**Figure 3:**
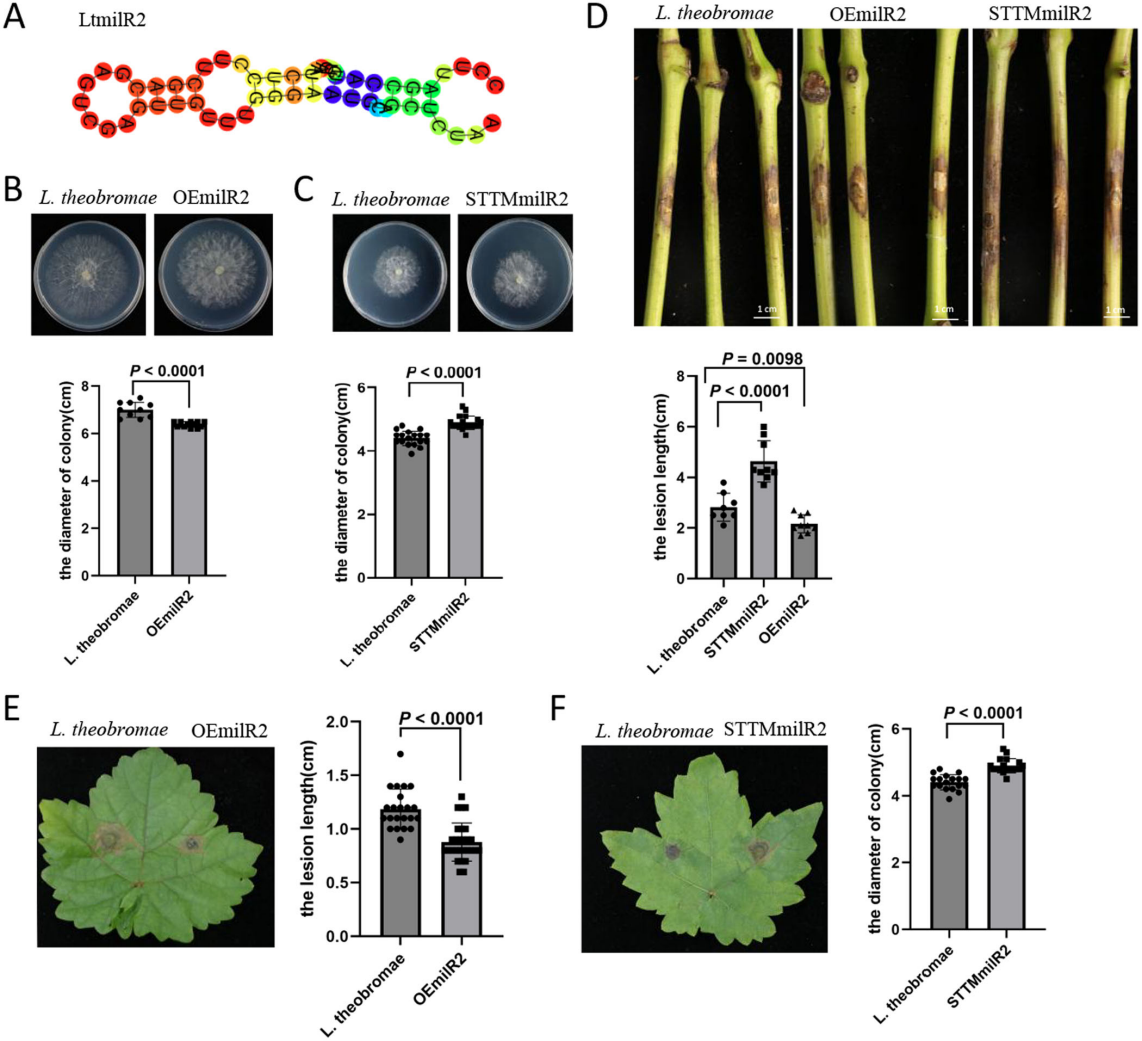
LtmilR2 negatively regulated the pathogenicity of *L. theobromae*. (A) Stem loop structure of LtmilR2. Colony morphology and diameters of OEmilR2 (B) and STTMmilR2 (C) after 24 h growth in PDA medium. Colony diameter was measured (*n* > 8), and statistical analysis was conducted using two‐sided *t*‐test. (D) Pathogenicity assay of OEmilR2 and STTMmilR2 infected on detached bark of *Vitis vinifera* cv. Summer Black. Lesion size was measured 3 days post‐inoculation (*n* > 10), with significance assessed by two‐sided *t*‐test. Pathogenicity assay of OEmilR2 (E) and STTMmilR2 (F) infected on detached leaves of *Vitis vinifera* cv. Summer Black. Lesion size was measured 3 days post‐inoculation (*n* > 10), with significance assessed by two‐sided *t*‐test. [Color figure can be viewed at wileyonlinelibrary.com]

**Figure 4:**
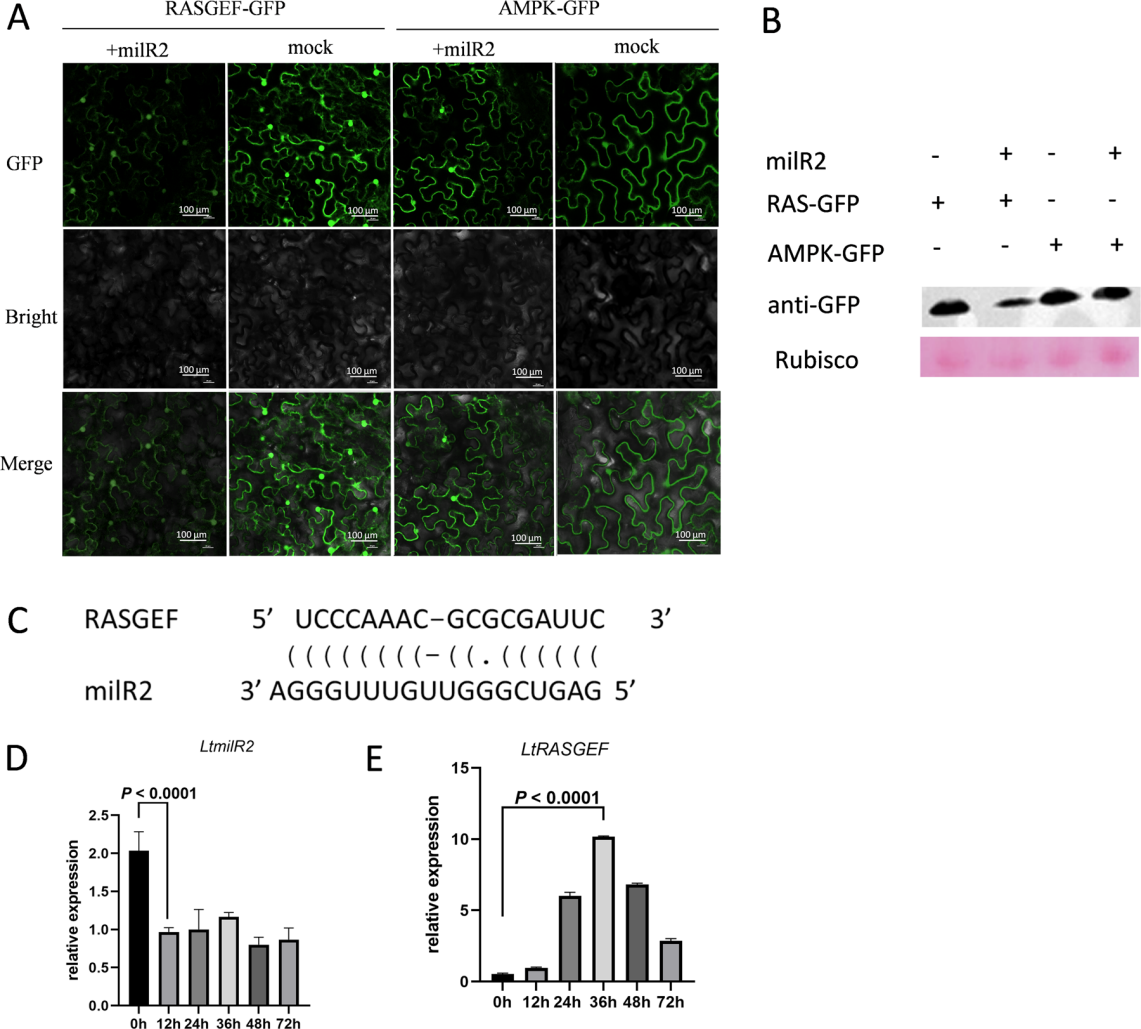
LtmilR2 targeted *LtRASGEF* at the transcriptional level during infection. (A) Confocal fluorescence microscopic images of transiently expressing RASGEF‐GFP/AMPK‐GFP with or without LtmilR2. The scar bar represents. (B) Western blot analysis of GFP expression level in A. (C) the target site of LtmilR2 to RASGEF. (D) The expression level of LtmilR2 during the infection by stem loop RT‐PCR and statistical analysis was conducted using two‐sided *t*‐test. (E) The expression level of *LtRASGEF* during the infection by RT‐PCR and statistical analysis was conducted using two‐sided *t*‐test. Data are means ± SD for three replicates. [Color figure can be viewed at wileyonlinelibrary.com]

Both *LtAGO1‐23* and *LtAGO1‐24* mutants exhibited no significant differences in colony morphology compared to the wild type; however, mycelial growth was accelerated (Figure 1A,B). After 48 h of growth on PDA plates supplemented with 0.05% H_2_O_2_, the *LtAGO1‐23* and *LtAGO1‐24* mutants showed no observable differences from the wild type (Supporting Information S1: Figure 2A,B). Under salt stress, however, the growth rate of *LtAGO1* mutants decreased on 0.5 M KCl, although colony morphology remained unchanged (Supporting Information S1: Figure 3A,B). Additionally, *LtAGO1* mutants displayed significantly more branched mycelia compared to the wild type (Supporting Information S1: Figure 4A), though there was no significant difference in overall mycelial biomass between mutants and wild type (Supporting Information S1: Figure 4B). For pathogenicity, *LtAGO1‐23* and *LtAGO1‐24* inoculated on grape leaves produced significantly larger lesions than the wild type, suggesting that *LtAGO1* mutants exhibit enhanced virulence (Figure 1C,D).

To investigate how *LtAGO1* regulates gene expression and thereby negatively affects pathogenicity, we conducted transcriptome analysis of the *LtAGO1‐23* mutants. In comparison with the wild type, 237 genes were significantly downregulated, while 37 genes were significantly upregulated in the *LtAGO1‐23* mutant after 24 h of infection (Figure 1E). GO enrichment analysis indicated that the genes with significant changes were primarily associated with xyloglucan metabolism, mycotoxin biosynthesis and fungal cell wall organization (Figure 1F). Notably, the degradation of plant xyloglucan is considered essential for successful host infection by pathogenic fungi (Lagaert et al. 2009).

### 2.2 LtmilR2 as an AGO1‐Dependent milRNA Negatively Regulates Virulence

**Figure 5:**
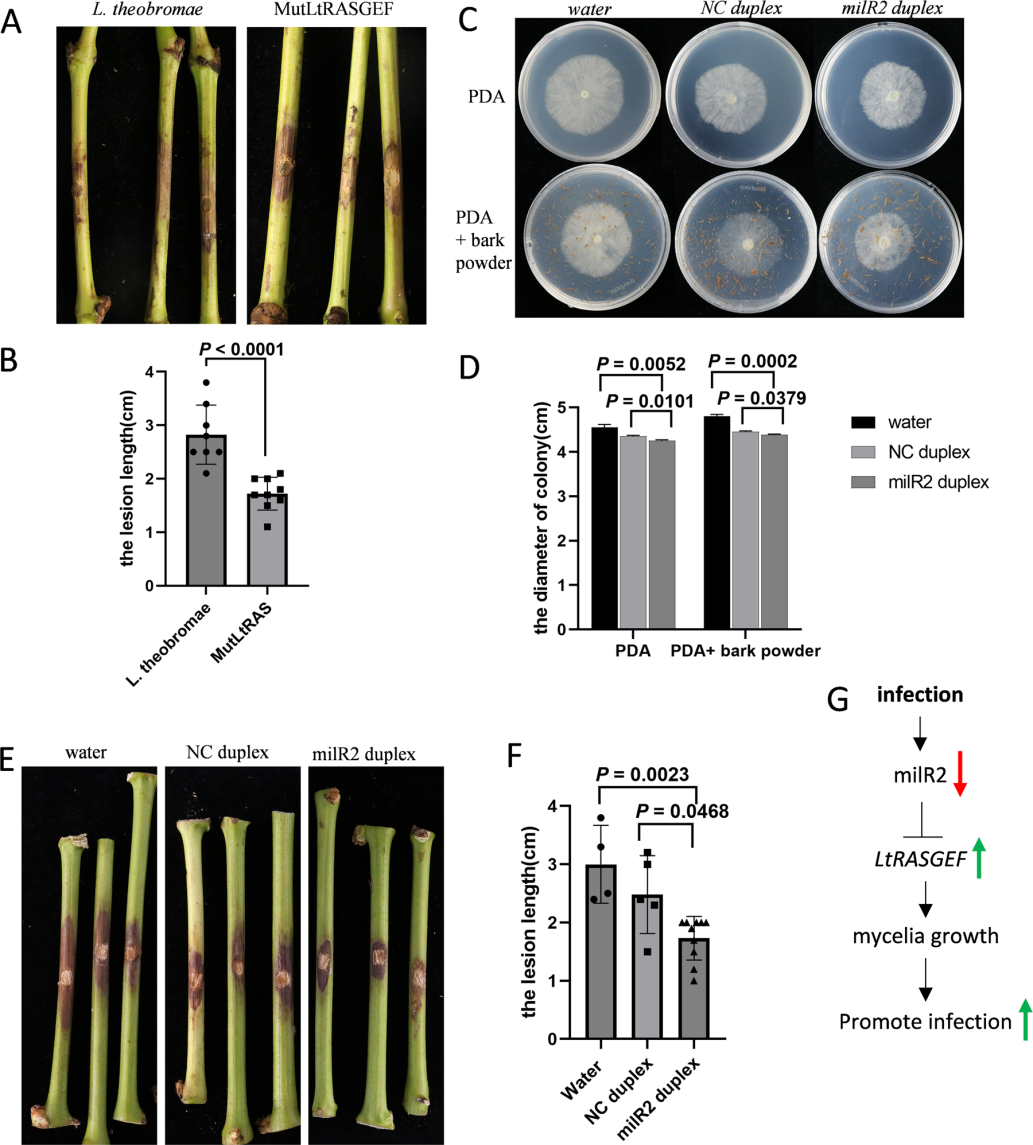
LtRASGEF positively regulated the pathogenicity of *L. theobromae*. (A) Pathogenicity assay of MutRAS and *L. theobromae* infected on detached bark of *Vitis vinifera* cv. Summer Black. (B) Lesion size was measured 3 days post‐inoculation (*n* > 10), with significance assessed by two‐sided *t*‐test. (C) Colony morphology of OEmilR2 and STTMmilR2 after 24 h growth in PDA medium with or without exogenous LtmilR2 duplex. (D) Colony diameter was measured (*n* > 8), and statistical analysis was conducted using two‐sided *t*‐test. (E) Pathogenicity assay of *L. theobromae* infected with exogenous LtmilR2 duplex or NC duplex on detached leaves of *Vitis vinifera* cv. Summer Black. (F) Lesion size was measured 3 days post‐inoculation (*n* > 10), with significance assessed by two‐sided *t*‐test. (G) The model of LtmilR2 targeted *LtRASGEF* to positively regulate the pathogenicity of *L. theobromae*. [Color figure can be viewed at wileyonlinelibrary.com]

To further elucidate the mechanism underlying AGO1‐mediated pathogenicity, sRNA sequencing was performed on both the *LtAGO1‐23* mutant and wild‐type strains (Supporting Information S2: Table 2). Compared with the wild type, nine milRNAs were significantly downregulated (Supporting Information S2: Table 3). Structural analysis showed seven sRNAs form a typical miRNA stem‐loop structure (Supporting Information S1: Figure 5), suggesting they belong to the milRNA family. What is more, bioinformatic analysis showed the majority of sRNAs were concentrated at lengths of 20–22 nucleotides (nt) (Supporting Information S1: Figure 6A). Kyoto Encyclopedia of Genes and Genomes (KEGG) enrichment analysis revealed that the target genes of these milRNAs are primarily associated with pathways including pantothenic acid and coenzyme A biosynthesis, phenylalanine metabolism and astrocystin synthesis (Supporting Information S1: Figure 6B). By stem‐loop RT‐PCR, four milRNAs—LtmilR2, LtmilR3, LtmilR5 and LtmilR7—were confirmed to be significantly downregulated, indicating their dependence on the LtAGO1 pathway (Figure 2A). To assess the effects of these milRNAs on the pathogenicity of *L. theobromae*, transformants overexpressing each milRNA were generated (Supporting Information S1: Figure 7). Among these, LtmilR2‐overexpressing transformants exhibited significantly smaller lesion sizes compared to the wild‐type *L. theobromae* strain, indicating a reduction in virulence (Figure 2B,C).

### 2.3 LtmilR2 Negatively Regulates Virulence

The structure of LtmilR2 was predicted using an online tool, revealing a typical stem‐loop structure (Figure 3A). To investigate whether LtmilR2 affects the pathogenicity of *L. theobromae*, overexpression (OEmilR2) and knockout (STTMmilR2) transformants were generated (Supporting Information S1: Figure 8). Both transformants were assessed for growth and infection capabilities. Growth did not differ significantly between OEmilR2, STTMmilR2 and the wild‐type *L. theobromae* (Figure 3B,C). Interestingly, OEmilR2 exhibited smaller infection areas compared to the wild type, while STTMmilR2 showed larger infection areas on both grape bark and leaves (Figure 3D). Additionally, on grape leaves, OEmilR2 again showed reduced infection areas, whereas STTMmilR2 displayed significantly larger infection areas compared to the wild type (Figure 3E,F). All these results indicated that LtmilR2 plays a negative role in the virulence of *L. theobromae*.

### 2.4 LtmilR2 Targets the *LtRASGEF* Gene to Suppress Its Expression

To identify the LtmilR2‐targeted mRNAs, we performed degradome sequencing. Over 200 potential target genes of LtmilR2 were identified, with a GEF of RAS (Rat sarcoma) signalling (*RASGEF*) (*LTHEOB_12543*) and AMP‐activated protein kinase (*AMPK*) (*LTHEOB_2512*) selected for further analysis. Co‐expression experiments were performed in *N. benthamiana* leaves to confirm the suppressive effect of LtmilR2 on these genes. The results showed a significant decrease in GFP intensity when LtRASGEF‐GFP was co‐expressed with LtmilR2, compared to LtRASGEF‐GFP expression alone. In contrast, GFP intensity did not change when LtAMPK‐GFP was co‐expressed with LtmilR2, indicating that LtmilR2 specifically targets *LtRASGEF* (Figure 4A). The difference in GFP expression was also confirmed by western blot analysis (Figure 4B). The target site within LtRASGEF was predicted by degradome sequencing, as shown in Figure 4B. In order to analyze the relationship between *LtmilR2* and its target gene *LtRASGEF* during the infection, relative expression was tested using stem‐loop RT‐PCR and qRT‐PCR. *LtmilR2* has a low expression during 12, 24, 36 and 48 h. However, at 0 h, *LtmilR2* had a high expression level. Which means when infection starts, the expression of *LtmiR2* decreased (Figure 4D). For the target gene of *LtmilR2*, *LtRASGEF*, increased transcription levels were observed after infection. which indicated the negatively correlated expression of *LtmilR2* (Figure 4E).

### 2.5 *LtRASGEF* Mutant Leads to Reduced Pathogenicity

To investigate the role of *LtRASGEF* in the pathogenicity of *L. theobromae*, *LtRASGEF* knockout mutants (MutLtRAS) were generated (Supporting Information S1: Figure 9). After 3 days of infection, MutLtRAS exhibited significantly smaller lesion areas, indicating a marked reduction in pathogenicity (Figure 5A,B). These findings suggest that LtRASGEF plays a positive role in the pathogenicity of *L. theobromae*. To further explore the potential of targeting *LtRASGEF* for RNA‐based fungicides, single‐stranded RNA duplexes of LtmilR2 and a 24‐nt random sRNA as a negative control (NC) were synthesized and encapsulated using the star polycation (SPc) nanocarrier. This formulation aimed to simulate an RNA fungicide for *L. theobromae*. The results showed that the LtmilR2 duplex significantly inhibited hyphal growth compared to the NC treatment (Figure 5C,D). Additionally, LtmilR2 duplex significantly inhibited infection of *L. theobromae* compared to the NC treatment on both grape bark and leaves (Figure 5E,F and Supporting Information S1: Figure 10). Our findings indicate that LtmilR2 is a promising RNA fungicide target for managing *L. theobromae*.

## 3 Discussion

*L. theobromae* is known to colonize the phloem of grapevines as an endophyte without causing disease for extended periods. However, under certain conditions, this fungus can lead to grape canker disease, which poses a severe threat to vineyards (Félix et al. 2019; Pandey et al. 2024). Despite its notoriety as a plant pathogen, effective fungicide solutions for managing grapevine canker disease remain unavailable.

In animals and plants, AGO is a critical protein in post‐transcriptional regulation pathways, where sRNA molecules bind to AGO proteins to form the RNA‐induced silencing complex (RISC). The AGO protein acts as a guide, directing the RISC complex to cleave the mRNA of target genes, resulting in inhibited translation, ultimately influencing gene expression at the transcriptional or post‐transcriptional level (Kim et al. 2010). However, in the model fungus *N. crassa*, four types of milRNAs were generated from four different pathways. Including Dicer, Quelling Deficient 2 (QDE2), QDE2 interacting protein (QIP) and MRPL3 (RNAse III domain‐containing protein). QDE2 is one of the two AGO proteins identified in *N. crassa*. Moreover, the most abundant milRNAs and maturation of milRNAs require AGO protein QDE2 (Chang et al. 2012; Xue et al. 2012; Lee et al. 2010). In this study, we found that *LtAGO1* negatively regulated the virulence of *L. theobromae*, indicating that post‐transcriptional regulatory mechanisms play a role in the pathogenicity regulation of this fungus.

Whether milRNA (sRNA) participates in the pathogenicity regulation of *L. theobromae* remains unknown. LtmilR2 was found to be an LtAGO1‐dependent milRNA that negatively regulated the virulence of *L. theobromae*. Using GFP co‐expression systems in *N. benthamiana*, a GEF of RAS signal was confirmed to interact with LtmilR2.

RAS proteins belong to guanine nucleotide‐binding protein (GTPase or G‐protein) that are present in all eukaryotes and participate in many biological processes (Hamm 1998; Harispe et al. 2008). Ras family members could cycle between GTP‐bound active state to GDP‐bound inactive state, which is regulated by GEFs and GTPase‐activating proteins (GAPs). GEFs promote the release of GDP from stable, whereas GAPs inactivate GTPases by hydrolyzing GTP to GDP (Bourne et al. 1990; Vetter and Wittinghofer 2001; Wennerberg et al. 2005). The Ras signalling pathway is critical for the virulence of many pathogenic fungi (Fortwendel 2012). Ras signalling was suggested as a key pathway to regulate sclerotia formation and virulence of *S. sclerotiorum* (Chen and Dickman 2005; Y. Xu et al. 2024). In this study, knockout RASGEF of *L. theobromae* resulted in decreased virulence. We propose a model for how LtmilR2 regulates the pathogenicity of *L. theobromae*. LtmilR2 has a high expression level in the mycelium. However, upon infection, the expression of LtmilR2 decreased significantly. As the gene expression of *LtRASGEF* was inhibited by LtmilR2. On the contrary, upon infection, the expression of *LtRASGEF* rapidly increased, enhancing the infection of *L. theobromae* (Figure 5G).

Based on the research conclusion, we aim to test whether milR2 could be used as an RNA fungicide target. LtmilR2 duplex is a double‐stranded sRNA designed based on the mature sequence of LtmilR2, including a sequence that is consistent with the mature LtmilR2 sequence, as well as a complementary sequence to the mature LtmilR2 sequence. The SPc‐based nano‐delivery system SPc is also used to enhance the function of the milRNA duplex (Su et al. 2023). Based on that, RNA duplex of LtmilR2 carried with SPc was designed and the results showed that it inhibited the growth of *L. theobromae* on PDA plates and also on the leaves. NC milR2 duplex also shows some inhibitory effect, this is consistent with a fact that several types of nano‐carriers could affect the activity of fungi, viruses and insects by themselves(Jiang et al. 2024; Yin et al. 2024; Yan et al. 2022). This indicates that LtmilR2, together with SPc, could be a RNA fungicide target of grape canker disease, a major threat to the grape industry. This RNA fungicide could be registered and replace the chemical fungicide for grapevine health. This is one of the major directions of future fungicide research.

## 4 Methods and Materials

### 4.1 Fungal Strains and Plants

The CSS‐01s (GenBank Number: GCA_002111425.1) strain of *L. theobromae* was screened and preserved in the lab (Yan et al. 2017). Branches and leaves of the grape variety ‘Summer Black’ from Xiangyi Vineyard, Shunyi, Beijing. Fungal strains were cultured on potato dextrose agar (PDA; 20% potato, 2% glucose and 1.5% agar) at 25°C. Liquid potato dextrose (PD) was used as the growth medium in the mycelium collection. Liquid PD with grape bark powder was used to mimic the infection.

### 4.2 Sequence Characterization and Phylogenetic Analysis

The *LtAGO1* sequences were isolated from the CSS‐01s genome database in NCBI BLAST website(https://blast.ncbi.nlm.nih.gov/Blast.cgi). Using the AGO sequences of *Homo sapiens* and *Arabidopsis thaliana* as probe sequences. The alignment of multiple protein sequences was done with DNAMAN software, and the phylogenetic tree was built using the neighbour‐joining methods in MEGA11(https://www.megasoftware.net/).

### 4.3 sRNA Sequencing and Data Analysis

Construction and sequencing of all sRNA libraries were done by Lc‐Bio Technologies (Hangzhou, Zhejiang, China). sRNA sequencing libraries were generated by TurSeq Small RNA Sample Prep Kits (Illunina, San Diego, USA). The library construction was performed with Illumina Hiseq2000/2500, and the sequencing read length was 1× 50 bp.

Adapter with dimers, junk, low complexity, common RNA families were removed. Subsequently, unique sequences with length in 18~26 nucleotide were mapped to specific species precursors in miRBase 22.1 by BLAST search to identify known miRNAs and novel 3p‐ and 5p‐derived miRNAs. The filtered data were considered to be the clean data.

### 4.4 Degradome Sequencing and Target Gene Prediction

The degradome libraries were constructed by Lc‐Bio Technologies. Sequencing was done by Illumina Hiseq2000/5000, generating single‐end reads with a read length of 1× 50 bp. CleaveLand procedure was used for analysis of degradation group (Addo‐Quaye, C. Bioinformatics, 130‐131).

Degradome sequencing specifically captures and sequences the ends of RNA fragments, particularly those generated by miRNA‐mediated cleavage. By using software CleaveLand v4.3, degradome sequencing identifies precise cleavage sites on mRNAs of *L. theobromae*. These sites indicate where miRNAs have bound and induced cleavage. Only targets with *p* < 0.05 and category < 4 in each sample were identified for discussion.

The miRNA target genes were also analyzed by the Gene Ontology Consortium (http://geneontology.org/) and the KEGG (http://www.genome.jp/kegg/). Gene Ontology functional enrichment and KEGG pathway analyses were performed on target genes of miRNA.

### 4.5 Generation of milRNA Overexpression and Knockout Transformants

The milRNA precursor was amplified from the genomic DNA of L. theobromae to create milRNA overexpression transformants. The amplified fragment was inserted into the pDL21 plasmid using the ClonExpress II One Step Cloning Kit (Vazyme Biotech, China). The construct was confirmed through sequencing to ensure the accuracy of the inserted sequence. The verified plasmid was then introduced into *L. theobromae* protoplasts using PEG‐mediated transformation. Transformants were screened and validated through stem‐loop qRT‐PCR, and those confirmed to express the construct were identified as OEmilRNA overexpression strains.

For the generation of milRNA knockout transformants, short tandem target mimic (STTM) sequences of LtmilRNA were designed based on Y. Wang et al. (2019), synthesized and cloned into the PDL2 vector by Sangon Biotech (China) (Y. Wang et al. 2019). The validated plasmid was transferred into *L. theobromae* protoplasts via homologous recombination. Transformants were verified by stem‐loop qRT‐PCR, and correctly validated strains were designated as STTMmilRNA strains.

### 4.6 Generation of LtRASGEF and LtAGO1 Mutants

The knockout transformants mainly involve amplifying upstream and downstream homologous fragments on both sides of *LtRASGEF/LtAGO1* separately. The amplified fragments were inserted into plasmid pKVOL21 by a double‐joint PCR. Sequencing was performed to verify the correction of the constructed vector. The correct plasmid was transferred into the protoplast of *L. theobromae* (He et al. 2024) by homologous recombination. Transformants need to be verified by qRT‐PCR. The correctly validated transformants were identified as strain MutLtRASGEF/LtAGO1.

### 4.7 Transfection of RNA Duplex Targeting *L. theobromae* LtmilR2

miRNA duplex (dsRNA, one strand is LtmilR2 mimics, and another is the complementary sequence which can enhance the stability of LtmilR2 mimic) as well as random dsRNA as a NC were designed and synthesized through the company (GenePharma). In total, 100 nM miRNA duplex oligomers were spread on prepared PDA plates or grape leaves (18 h/6 h day/night; 25°C). After 2 days of incubation, the diameter of colony was measured. After 3 days, lesions were recorded on grape leaves and the virulence of different treatments was measured.

### 4.8 Vegetative Growth and Pathogenicity of milRNA Mutants of *L. theobromae*

The milRNA mutants and wild‐type strains were activated on PDA plates and incubated at 25°C for 2 days. Mycelium plugs (5 mm in diameter) from the edge of growing colonies were then inoculated onto PDA. The plate was cultivated at 25°C in the dark, 24 h, 48 h and 72 h, and finally until the mycelium grows all over the Petri dish, used for the observation of the morphology of the colony. The data were finally analyzed by GraphPad Prism for the significance of differences.

The inoculation test was performed on detached *Vitis vinifera* cv. Summer Black green shoots with methods described in (Harishchandra et al. 2020) or on the back of *N. benthamiana* leaves that had been stabbed with sterilized insect needles. The mycelium plugs were placed on the wound with the hyphae face down, and each *N. benthamiana* leaf was set as a replicate, with a total of 30 replicates, and the blank PDA medium was used as a control. The inoculated tissues were cultured in a greenhouse with 25°C light, 18°C darkness, 12 h light–dark alternation and 80% relative humidity.

Susceptibility assay: 5 mm mycelium plugs of the milRNA mutant strain and wild‐type mutant strain were inoculated onto PDA plates with 0.05% H_2_O_2_ and 0.5 M KCl, respectively. Then they were placed in a light incubator at 25°C for incubation. The colony morphology and size were checked after 1–2 days.

### 4.9 milRNA Stem‐Loop qRT‐PCR

Total RNA extraction was conducted using the miRute milRNA Extraction and Isolation Kit (Tiangen, Beijing, China). The expression analysis of milRNAs was performed using the stem‐loop qRT‐PCR quantification method, as described previously (Varkonyi‐Gasic et al. 2007). In total, 2 μg first‐strand cDNA synthesis was carried out using the milRNA First Strand cDNA Synthesis Kit (Sangon Biotech, Shanghai, China) and stem‐loop RT primers, following the manufacturer's instructions. PCR amplification utilized specific forward primers paired with universal reverse primers. Nuclear RNA U6 from *L. theobromae* served as the internal control for normalization and quantification. All primers were listed in Supporting Information S2: Table 1.

### 4.10 Validation of the Targeting Relationship Between milRNA and Target Genes

To validate the interaction between the milRNA and its target genes (Supporting Information S2: Table 4), the precursor sequence of the milRNA and the target gene sequence containing the predicted binding region were amplified separately. Each amplified sequence was cloned into the pCAMBIA1301 expression vector, resulting in the constructs pCAMBIA1301‐pri‐milRNA and pCAMBIA1301‐target gene fragment‐GFP. These vectors were transformed into *Agrobacterium tumefaciens* strain GV3101. *Agrobacterium*‐mediated transient expression was conducted in *N. benthamiana* leaves. The GFP fluorescence signal in infiltrated leaves was monitored using a fluorescence microscope (LSM900, Zeiss, Germany). To assess the effect of milRNA on the expression of the target gene. A decrease in GFP fluorescence intensity would indicate successful targeting and suppression of the target gene by the milRNA.

## Conflicts of Interest

The authors declare no conflicts of interest.

## Supporting information

Support figure‐revised vcv.

Supporting table 1‐4‐revised.

## Data Availability

The data that support the findings of this study are available on request from the corresponding author. The data are not publicly available due to privacy or ethical restrictions.
