# Supplementary material for: Argonaute1‐Dependent LtmilR2 Negatively Regulated Infection of Lasiodiplodia theobromae by Targeting a Guanine Nucleotide Exchange Factor in RAS Signalling
Source: Plant Cell Environ. 2025 Jul 13;48(10):7688–98. doi: 10.1111/pce.70058 (PMC12415416; doi:10.1111/pce.70058)
Supplement: Supplementary file 1 — Support figure‐revised vcv. [file PCE-48-7688-s002.pdf]

Figure S1

A

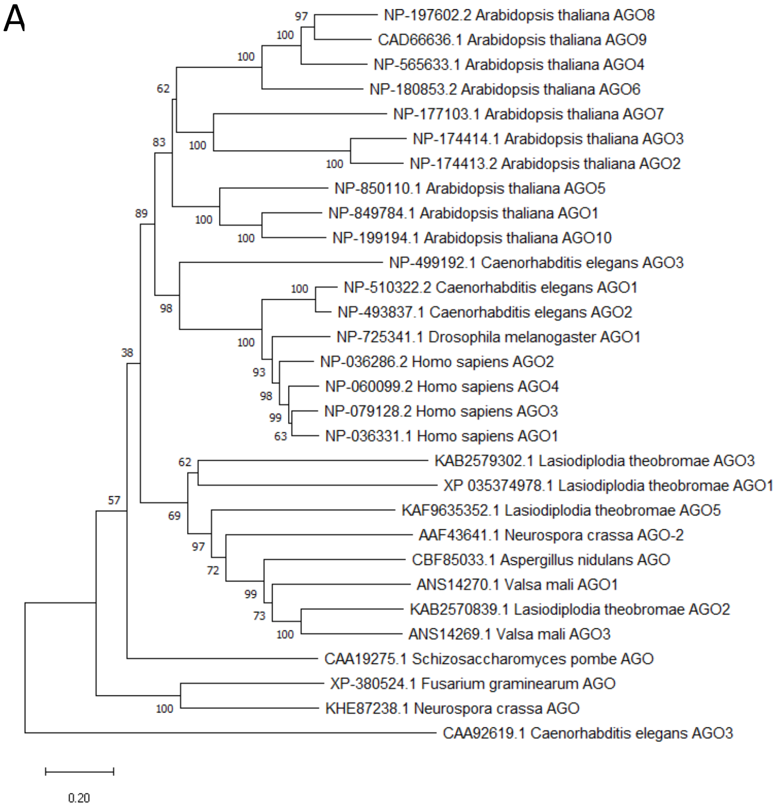

B

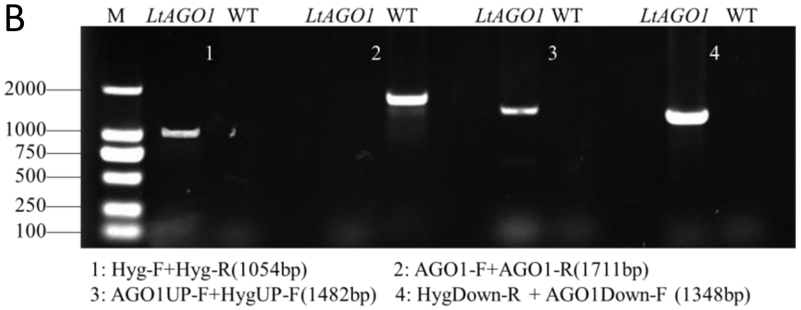

Figure S1. (A) Phylogenetic analysis of four AGO proteins of *L. theobromae*. (B) *LtAGO1* (#23) mutant was confirmed by four pairs of primers by PCR.

Figure S2

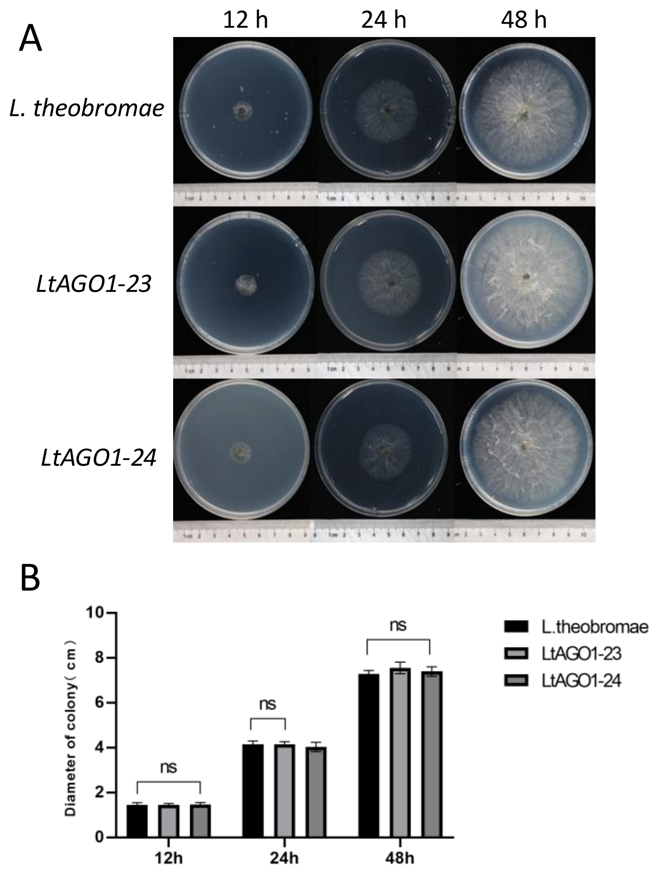

Figure S2. (A) Colony morphology and diameter analysis of *LtAGO1* (#23 and #24) under reactive oxygen species stress (0.05% H_2O_2). (B) Statistical analysis (n > 8) of colony diameter under reactive oxygen species stress (0.05% H_2O_2), with significance assessed by t- test (ns p >= 0.05).

Figure S3

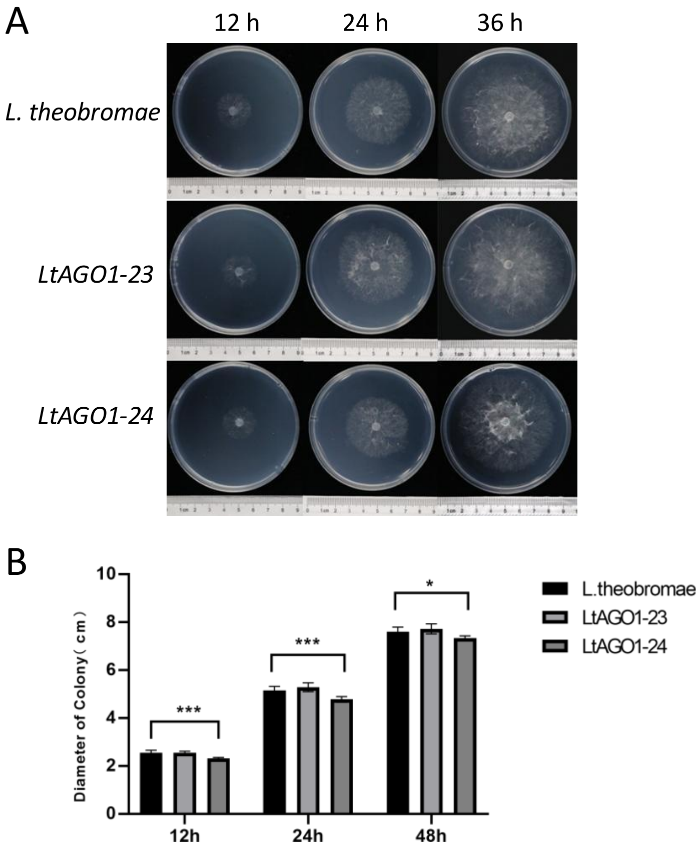

Figure S3. (A) Colony morphology and diameter analysis of *LtAGO1* (#23 and #24) under osmotic stress (0.5M KCl). (B) Statistical analysis (n > 8) of colony diameter under osmotic stress (0.5M KCl) test (*** $p < 0.001$, * $p < 0.05$) .

Figure S4

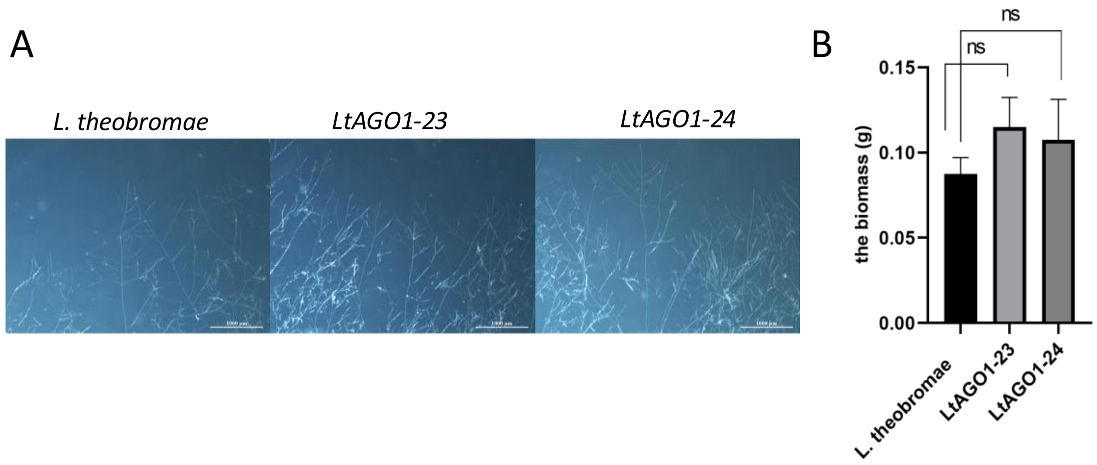

Figure S4. (a) mycelial morphology of *LtAGO1*(#23 and #24) after 48 hours of growth on PDA medium. (b) the biomass of *LtAGO1* (#23 and #24) after 48 hours of growth on PDA medium, significance assessed by t -test (ns $p >= 0.05$).

Figure S5

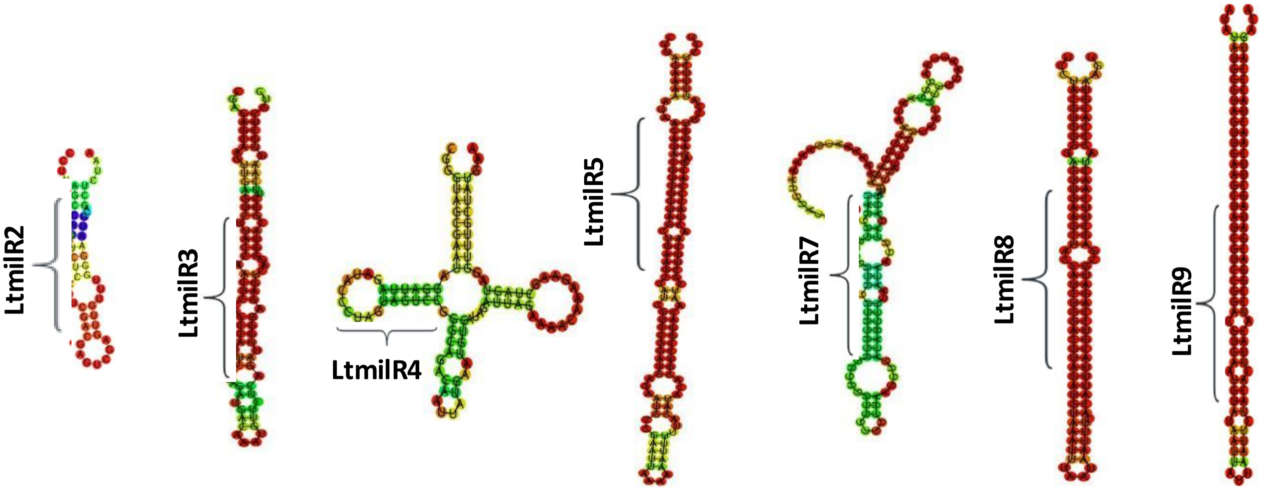

Figure S5. The Structural analysis of candidate AGO1-dependent milRNAs forms a typical miRNA stem-loop structure.

Figure S6

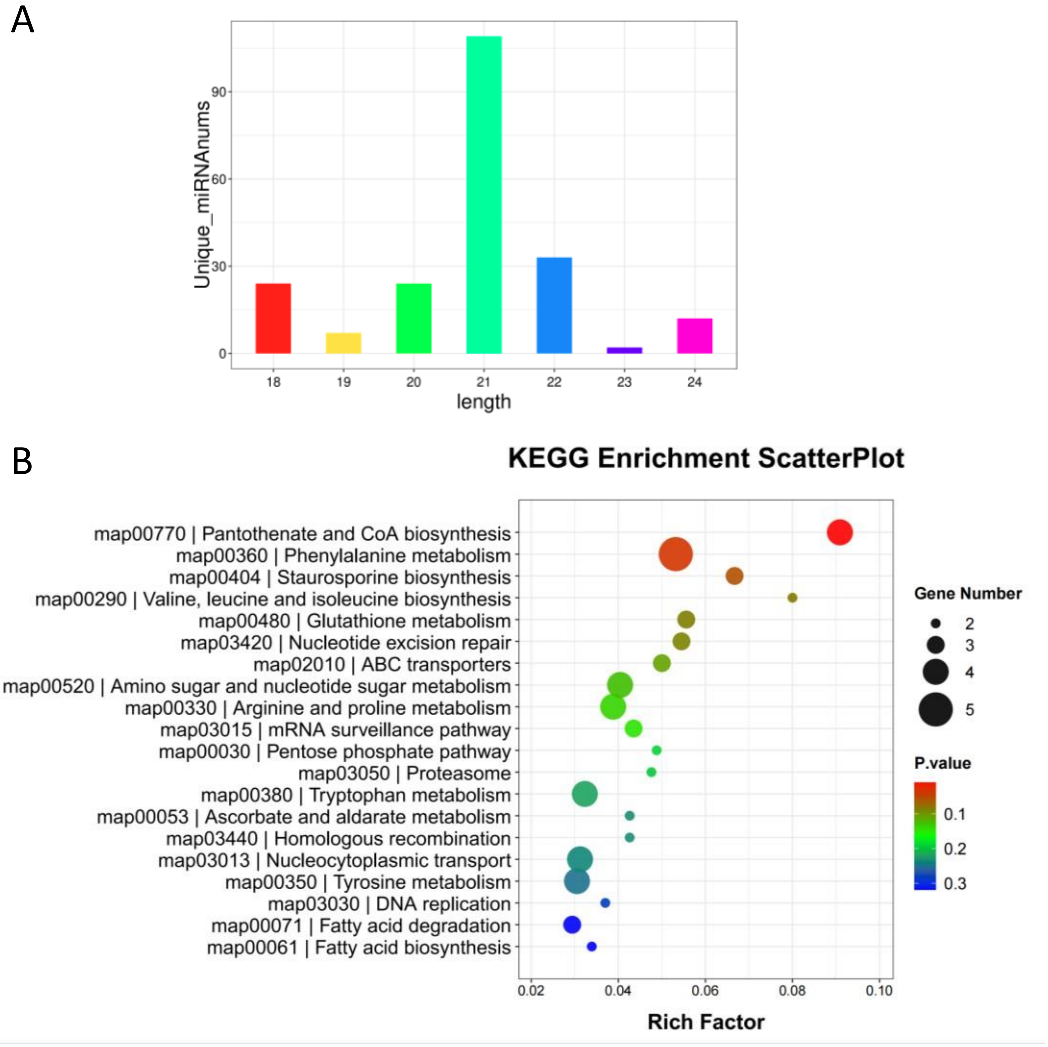

Figure S6. (A) the distribution of the length of miRNAs.(B) KEGG enrichment analysis of target genes of candidate milRNAs

Figure S7

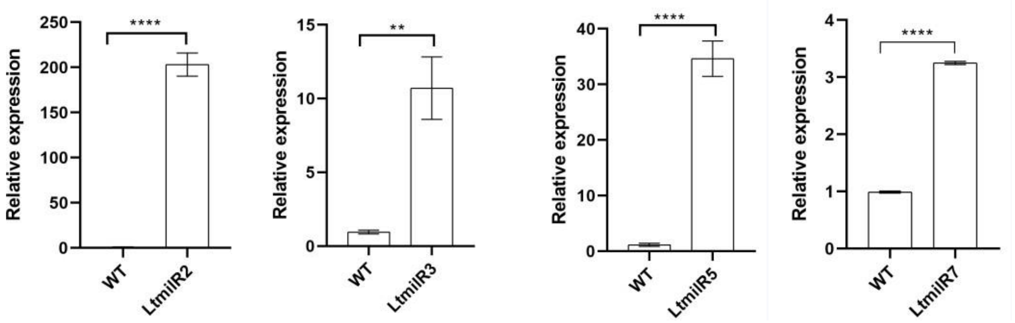

Figure S7. The expression analysis of OEmilR2, OEmilR3, OEmilR5, OEmilR7 by stem loop RT-PCR and statistical analysis was conducted using t -test (**** $p < 0.0001$, ** $p < 0.01$).

Figure S8

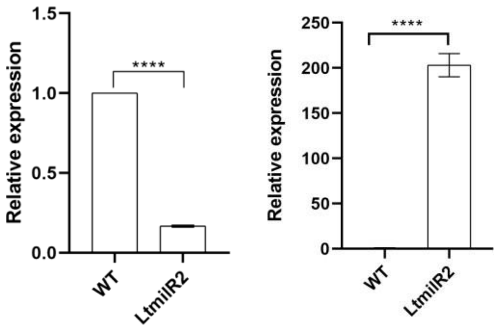

Figure S8. The expression analysis of OEmilR2 and STTMmilR2 by stem loop RT-PCR and statistical analysis was conducted using t -test (**** $p < 0.0001$).

Figure S9

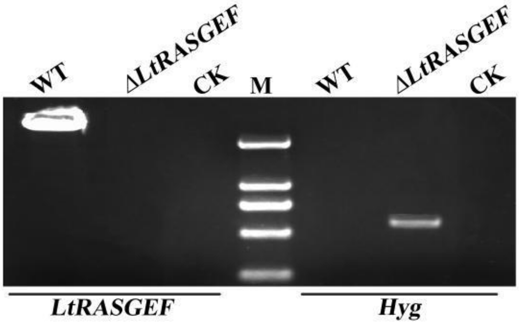

Figure S9. The RASGEF mutant was confirmed by four pairs of primers by PCR.

Figure S10

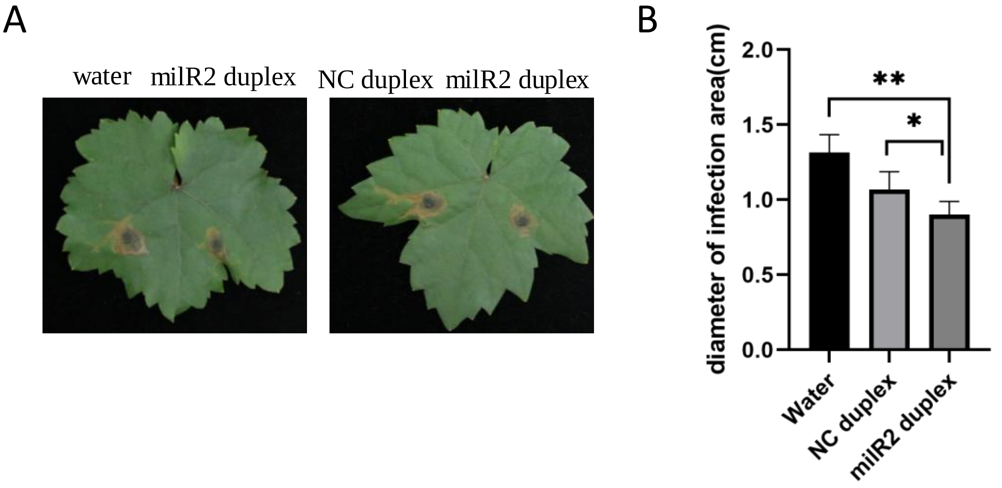

Figure S10. (A) Pathogenicity assay of *L. theobromae* infected with exogenous LtmilR2 duplex or NC duplex on detached leaves of Vitis vinifera cv. Summer Black. (B) Lesion size was measured 3 days post-inoculation ($n > 10$), with significance assessed by t- test (**$p < 0.01$, *$p < 0.05$).
